# Factory Commission

**Published:** 1834-04-01

**Authors:** 


### FACTORY COMMISSION.

1. Instructions from the Central Board of Factory Commissioners to the District Medical Commissioners.
2. Reports by the Medical Commissioners, Nos. 1, 2, 3, 4.

Could the production of sugar have been continued in the West Indies, and that of calico at home, without attracting the notice of humanity to the means employed—or were there no medium existing through which the horrors of these means could be presented to the public eye, many a revolving year would have passed ere we should have heard (if, indeed, we ever should) of the Slave-Trade-Abolition Bill, or the appointment of a Factory Commission. The Treasury would have gone on annually to receive, and pour into the lap of its thousand dependants, the golden contributions of these abundant springs of national wealth; Ministers would have boasted in Parliament, nay, Majesty itself would have proclaimed from the throne, the progressive prosperity of our colonies and manufactories, without, however, wounding public or individual sensibility, by alluding to the amount of human suffering necessary to furnish the materials for so bright a picture. But the press was there, that irresistible engine of public opinion—that palladium of social liberty and social advancement—that mighty power, unknown to antiquity, which ensures freedom from oppression wherever it is itself free. The mild, impressive pleadings of the pure philanthropist, the hypocritical groans of the saintly sinner, the oratory of the selfish agitator, all found a fertile theme in the wrongs of the expatriated colonial slave, or of the overworked factory child nearer home—all found a ready organ in the public press to melt their complainings into unison, and to make them heard wherever civilized man is found. Forthwith, the greedy proprietor, the calculating financier, the cold-blooded Malthusian, were compelled to yield to the universal ebullition of the best of human feelings; and the immediate consequence was, the emancipation of our colonial slaves—the Twenty-millions-Bill, and (what especially forms the subject of the present article) the legislative measures recently adopted, protective of infant health and infant labour.

Discussion as to the commercial policy of these measures, the influence which they may exercise upon the morals, the wealth, and the peace of the country, would be foreign to the purposes of our Journal. We hold ourselves bound to watch the progress of medical science only, and to lay before the profession, from time to time, every new and interesting fact with which that science is concerned. In the present instance, knowing that a Royal Commission was issued on the 19th of April, 1833, appointing, with others, five medical gentlemen of eminence to investigate the effects of factory labour upon the health of those so employed, but more particularly upon that of children, it becomes our duty to remark upon the organization, the mode of conducting, and the Reports of that Commission.

### Organization and Distribution of the Commission.

Four districts having been traced out, comprehending the seats of the principal branches of manufacture in which any large proportion of infant labour is employed, the ten civil, and five medical gentlemen composing the general Factory Commission were divided into five lesser commissions, each having one medical, and two civil members, as follows:—

1. A Central Commission, or Board, composed of a chairman, Mr. Tooke, distinguished as a writer on currency, and as an enlightened political economist —Mr. Chadwick, already a most efficient member of the Poor-Law Commission —Dr. Thomas Southwood Smith, an eminent physiologist, and author of an Essay on Fever, remarkable for the talent and research it displays. This Commission held its sittings in Whitehall Yard, never quitted London, framed instructions for the District Commissioners, and prepared the general report.

2. The Commission for Scotland and the North of Ireland, consisting of Mr. Stuart, the accurate and independent author of Travels in America—of Mr. Mackintosh, a young Barrister, son of the late eminent orator and statesman of that name, a large inheritor of his father's high talents and liberal views—and of Sir David Barry, author of Experiments on the Circulation of the Blood, and member of the late Central Board of Health.

3. The Medical Commissioner for the North-eastern District of England, was Dr. Loudon of Leamington, and with him were united Messrs. Drinkwater and Power.

4. The Lancashire District fell to the lot of Dr. Bisset Hawkins, author of "Medical Statistics," with Mr. Cowell and Mr. Tufnell.

5. Dr. Woolriche, Inspector General of Army Hospitals, was named for the

Western District, having for his collaborateurs Mr. Spencer and Mr. Leonard Horner, whose name is so intimately connected with the early progress of the London University.

Although, by the fact of having appointed a Factory Commission at all, Ministers incurred much obloquy, all of which they might have avoided by legislating at once, upon the evidence adduced by Lord Ashley and Mr. Sadler; yet there can now be very little doubt, that in waiting for the results of local inquiry they pursued the wisest and fairest course, more especially as they had that inquiry conducted by disinterested professional men, who spared no pains to come at the truth.

### Instructions.

The only instruction received by the Central Board, independently of the terms of the Commission itself, was a verbal intimation, that the inquiry was expected to be "into the whole truth respecting the employment of children in factories." The detailed instructions issued by this Board to the District Medical Commissioners, occupying 35 quarto pages, with numerous and complicated tables and queries to be filled up and answered,.must be considered as having emanated from the Central Medical Commissioner. He alone, therefore, must abide the consequences of their examination. Whatever faults or perfections these instructions, as a whole, may be found to possess, they certainly are open to the following objections:—

1st. The utter impossibility of executing certain portions of them.

2dly. The evident tendency of these portions to bring down obloquy and ridicule on the whole Commission, and on the medical profession generally. We allude to the tabular questions at pages 24 and 26 of the Medical Instructions; of which the following are examples:—

" Quest. 8. Was your first child born within one year of your marriage?

" 10. How many miscarriages? in the first three months—in the next three months."——" In the last three months of pregnancy."

" 11. How many of the births were difficult cases; requiring instruments—not requiring instruments?"

" 12. What is the name of the father of your child or children?"

" *State how many weeks in each of the following years of age each child has been sick—1st year, 2d year, 3d year, 4th year, 5th year.*"

When we learn that these and many other such questions were to be put, not only to all married women employed in factories, but likewise to at least an equal number of married women not so employed; that a table constructed upon the answers was to be filled up for each woman, and for each child whether dead or living; and that all this, besides the really useful duties of the medical gentlemen, was to be executed between the 24th of April and the end of June; there can be no exaggeration in pronouncing the task impossible, and the labour and expense useless, which was bestowed on its construction. Indeed, Dr. Smith himself says to the District Medical Commissioners, at p. 16 of his Instructions,—" That in the short space of time allotted to the execution of the Commission, you should be able, by your own personal inquiries, to obtain the information now sought for, is impossible;" and then suggests the following plan for its accomplishment. "In several of the large cities which you will visit there are medical schools." "A few medical pupils, two by two, taking a street and making the inquiries from house to house, would speedily and effectually accomplish the task. In the hope that this suggestion may be carried into effect the Tables of Inquiries to be made of married women have been made up into books containing *fifty each*, which may be distributed to each medical man, or medical student, in order that he may fill up the set."

What are we to say to the medical ethics of boys thus employed to cross-examine matrons upon such points?

So glaring was the unfitness of these questions to the purposes of the inquiry, and so strongly did they outrage public feeling at the time, that the eulogist of the Government felt himself called upon to visit them with a deprecating sentence or two in his eloquent exposé of "What have the Ministers done?" These are the questions also which, with their tables attached, were printed and issued to the District Commissioners in such prodigious masses, that the post in Scotland, as we learn from high authority, absolutely broke down under their weight, and thereby interrupted the correspondence of the country for several hours. But after all, the best comment upon these impracticable and purely theoretical instructions is, that of the cartloads of blank printed tables and queries transmitted to the District Medical Commissioners, not even one solitary, filled up specimen appears in any of the Reports.

This attempt to tack on a theoretical scheme of general statistics upon a specific, practical investigation was ill-judged, to say the least of it, and might have been fatal to the success of the Commission. But on such occasions as these, the proper objects of inquiry are too plain to be mistaken, and indeed almost all instructions are fortunately compressible into a small compass; whatever may be the extent of paper over which they are spread. In the present case all that was essential in the thirty-five pages already mentioned, may be found in the three following sentences or headings, which might with advantage have been condensed into one. The Medical Commissioners are told to investigate—

1. "The actual condition of the manufacturing population (including children and adults) relatively to health and disease." p. 7.
2. "The present condition of the manufacturing population (including children and adults) compared with its condition at former periods." 12.
3. "Comparative state of the children of the same age and class, in the same district, not factory children." 13.

The object which the locomotive Commissioners felt themselves obliged to keep in view, and that to which the terms of their commission expressly bound them was, "to collect information as to the employment of children in factories, and as to the propriety of curtailing their hours of labour." This brief instruction inculcates, so far as the Medical Commissioners were concerned, the necessity of ascertaining the effects of factory labour upon the health and growth of children so employed, with the view of suggesting remedies for such evils and abuses as might be found to exist in the present mode of conducting such labour.

We shall now take our leave of the official instructions, and proceed to examine the methods actually adopted by each of the four District Medical Commissioners, in conducting their inquiries, with the facts elicited by these gentlemen, as set forth in their respective Reports.

### Mode of Conducting the Inquiry—Reports of the Medical Commissioners.

At the head of the Medical Reports stands that of Sir David Barry. This gentleman appears not only to have spared no labour to obtain such information as might lead him to form just conclusions for himself, but to have placed that information before the Central Board in such circumstantial abundance, and in such perspicuous order as to enable them to judge whether his conclusions were or were not fairly deduced from the facts of the investigation. He first designates the individual factory, and then describes its locality, neighbourhood, elevation, and form of the buildings; next the interior; the height, temperature and ventilation of the working rooms; the drainage, state of cleanliness, and general atmosphere; sanitary precautions adopted from time to time, and the improvements made, tending to promote health, since the establishment of the factory. The next heads we find are clothing; its change on quitting the factory in Winter—hours of beginning, remitting, and leaving off work each day—cleansing machinery—holidays in the year—Sunday's employment of time—moral instruction—food and drink at and between meals—health of operatives generally—appearance—branch of manufactory least healthful—annual mortality (average for ten years)—usual diseases—what months most sickly—cholera (cases, deaths)—accidents from machinery—abortions—medical assistance. Next comes the classification of the operatives of each mill, under the following heads. Total number—males, females—under 15, above 15—married—single—Irish—natives. We have then "the individual, personal examinations of mill-workers," which Sir D. thinks ought to form the chief object of the Medical Commissioner—description of and sanitary differences between the various kinds of factory employment—comparison with other working classes, such as hand-loom weavers and colliers, which are also carefully investigated and described.

The indefatigable zeal and labour brought to bear upon the inquiry in the northern district were very remarkable. Not only do we find the general headings already mentioned carefully filled up; but the individual workers examined for physical defects, much after the fashion of military recruits; and lest any attempt should be made to keep the deformed or the maimed out of sight, the oldest overlookers and workers were publicly sworn, as to whether any, or how many such persons were actually at the time, or had recently been borne on the books of the establishment—and also whether they knew of any individual having become deformed during their employment at factory-work, no matter from what cause.

We cannot conceive how truth could be sought for in such a question with a fairer prospect of impartial results than by the method just described. The whole Report occupies 76 folio pages. Many of the facts stated are highly curious in themselves, and must prove so interesting to the medical reader, that we shall make no apology for quoting a few of them in the words of the original. In the Medical Report on Mr. Craig's flax spinning mill, at Preston Holme, employing 150 workers, 103 of whom were under 18, we find the following:—

"The diet consists, for breakfast at nine o'clock, of porridge, and milk when the latter is plenty; for dinner, at half past two o'clock, potatoes, broth, bacon, garden stuff—butcher's meat *seldom*; for supper, at nine o'clock, porridge or broth; and for drink at and between meals, water.

The health of the operatives in general appears excellent. Some few look rather delicate, but seem to work cheerfully. No foul tongues. Heard no coughing during several hours passed in working rooms. The appearance of by far the greater number was healthful, robust, fully grown for age. Did not see even one case of distortion or narrow pelvis. Many of the girls were beautifully formed, who had been from ten years to maturity in the mill. The branches of manufactory least healthful are the carding and heckling departments. No death has occurred among the workpeople during the last three years. The spring months are the most sickly. The usual diseases are colds and the ordinary diseases of childhood. Informed by Dr. Lucas, who happened to come to the village to see a patient, that the epidemics of the country, such as cholera, influenza, and scarlatina now prevailing generally, are milder in Preston Holme than in the neighbouring collier villages. There have been no deaths from scarlatina as yet there, but many in the other villages. Saw three mild cases myself."

"Dr. Stephenson, who has been the chief accoucheur to the factory women for the last fifteen years, has not had a single forceps case amongst them, although in that time he has attended upwards of thirty first labours. Abortion is not more frequent than amongst other women. There has been one illegitimate child within the last three years."

"With regard to the physical appearance of the young persons. I went round the village whilst they were at dinner, and saw no squalid, emaciated, nor stunted individuals. I noticed five sisters from thirteen upwards, all employed in the mill from their childhood, every one of whom might be termed a fine grown girl; some of them remarkable for symmetry and strength. The succession crop of infants in the village were very numerous and healthy-looking; generally destined for the mill." 2.

Here the author, as if doubting the evidence of his own senses, and apologizing to himself for not having found any thing at all approaching to his own unfavourable prepossessions, concludes his first report thus:—

"The very favourable situation of this mill, and the parental attention of Mr. and Mrs. Craig to the health and education of the children, render it a specimen of factory life which I fear we shall find to be very superior to the general average." 2.

The description of the wet spinning flax-mills at Dunfermline, in which the thread is made to pass through hot water, presents a very different picture of factory labour.

"Visited the mills mentioned in the accompanying reports. Find that thirteen and a quarter hours are the daily term of factory labour here, independently of meal-times; on Saturdays eleven hours.

The difference between the comfort, cleanliness, and apparent salubrity of the wet and dry spinning-mills is very strikingly in favour of the latter; more particularly when splash boards are not placed in front of the perns to protect the spinners from the spray. In the mills not protected the spinners are wet through early in the day about the abdomen, and present a dripping, draggled appearance."

"The spinners (almost exclusively girls from ten upwards) never sit down, or almost never, excepting at meal-times. A good spinner cannot be formed if she begin to learn later than eleven years of age. The reelers, who are the stoutest girls; the spreaders, also adults; the card-feeders—never sit; indeed there is nothing to sit upon; they sometimes lean for a few minutes against a frame. Yet taking all these classes, or any of them indiscriminately, their appearance is that of health and extreme activity. Many are finely formed and strikingly handsome, who have worked, as stated, from nine to maturity.

This day examined carefully and individually one hundred and eleven girls of the classes stated, with a view to find, if possible, a case in which the plantar-arch had been broken down by continued standing, as is stated in the evidence lately printed to occur sometimes in factory workers. Found many beautifully formed feet in those who had worked the longest. In one case, a woman, aged forty-three, who had worked from the age of seven, the foot was remarkably small and high in the instep. In no case did the plantar-arch seem to have been in the slightest degree disturbed."

"The pelves of the adult girls were remarkably well formed, with strikingly well-developed glutæi.

Nothing but the evidence of my own senses could have induced me to believe that girls, indeed any human beings, worked as stated from nine upwards, could yet possess, in maturity, the apparent extreme of high health and vigour, with finely-proportioned forms.

The wet spinning mills require amelioration. Wet clothes and bare feet on wet flagging, as in Kirkland's and Hall's, must produce disease. Half the spindles in the flagged rooms were idle in both mills from the indisposition of the workers; said to be influenza. Not so in the dry spinning-mills."

"Amongst those examined was one woman, Jannet Walls, aged forty, who had begun factory work when seven years old; she had been a spinner twenty-four years, she said; yet her feet and ankles were remarkably fine, but sinewy, and of sharp lean outline, like those of the male.

The medical men here, Dr. Douglas more particularly, are of opinion, that puberty in mill girls is apt to be protracted sometimes to the twentieth year. Dr. D. attends about 250 collier families, and states that their young people are more healthy than mill girls. Has never met a distorted or narrow pelvis in the latter during the seven years he has practised here; not one forceps case. Cholera more fatal amongst colliers. Wounds in mill-workers heal most kindly. Never heard of a case of tetanus in Dunfermline, nor has Dr. Bowes."

"From what I have been able to learn and observe, I consider the spinner as by far the most important operative in a flax-spinning mill, and the most difficult to be formed. The masters are unanimous in asserting that girls, and they alone are trained to flax-spinning, never become expert artists if they begin to learn after eleven. I observed two girls, for some time in Mr. Malcolm's mill, about thirteen each, in the same pass or space between two frames; one attended to sixty wet spindles, or the spinning of sixty threads of yarn, of five ounces to the hank, the other to fifty spindles.* The first had 11*d.* the other 10*d.* per day. The range which each girl had to move over along her spindles, or the length of the pass, was about twenty-two feet. It is quite impossible to give an adequate notion of the quickness and dexterity with which these girls joined their broken ends of threads; shifted the pirns; screwed and unscrewed the flies, &c. To supply the place of such artists by new adult hands would be utterly impracticable, and difficult in the extreme to find a relay of hands equally expert, under present circumstances. There is no sameness of attitude—no standing still; every muscle is in action, and that in quick succession." 5.

After having examined several hundred persons, of various ages, employed in flax-mills—after having observed them whilst at work at sundry hours of the day—during their hours of relaxation on Sundays and at their Sunday-schools, our Commissioner next gives a detailed, circumstantial account of the appearance, food, labour, and sanitary condition generally, of the individuals employed in twenty-three hand-loom weaving-shops, examined without selection, and then contrasts the conditions of the younger persons of both classes, by placing some of the more prominent circumstances connected with each in opposite *columns, thus :*—

| " *Young Weavers—Age from* 10 *upwards.* | *Young Mill-workers—from* 7 *or* 8 *upwards.* |
|---|---|
| 1. Sex.— Indifferently of either. | 1. Almost exclusively female. |
| 2. Posture whilst at work.— Always sitting. | 2. Always upright. |
| 3. Food, where taken.— Always at home. | 3. Very often in the mills, especially in large towns. |
| 4. Working room.— Always an earthen floor. | 4. Lower flats flagged; upper boarded. |
| 5. Temperature and atmosphere of working room.— Cool and agreeable in the summer, excepting when fermented potatoe dressing is used, which produces a most disagreeable smell. | 5. Generally hotter than surrounding atmosphere. In wet-spinning rooms very unpleasant smell: preparing rooms excessively dusty. |
| 6. Air and exercise.— Sometimes employed in the fields in planting their potatoes, and in collecting them; sometimes in getting in the harvest. | 6. Never employed in the field nor in the open air, except going to their meals, and on Sundays. |
| 7. Reading and writing.— Little or no time for either, except through permission from their parents. | 7. Can only be learned after working hours. |
| 8. Instruction in domestic duties.— Always under the eyes of their parents. | 8. Never but when they go home to meals; often only when they go home to sleep. |
| 9. In moral conduct.— | |

* " Each spindle produces, upon an average of days, half a spinal, that is two hanks (of twelve cutts and of five ounces of yarn each hank per day, according to Mr. Ayton's (of Kirkaldy) estimate. So that one girl now spins as much as 100 good hand-spinners per day formerly."

| | |
|---|---|
| The girls are watched over by their mothers. | 9. Never see their mothers but at meal-times. |
| 10. Amount earned.— Miserably small. In the case of Janet Anderson, only 1*s*. 5*d*. per week. | 10. Considerable—from 5*s*. to 6*s*. 6*d*. per week as a spinner, say of thirteen or fourteen years of age. |
| 11. Application of earnings.— United to those of the father, and other members of family, for the joint support of all. | 11. Often applied to the support of widowed parents and infant brothers and sisters; often wasted by idle father; sometimes in great part spent by girl herself (when not under her mother's control) in finery, while she stints herself in food. |
| 12. Sources of unhealthiness.— Confinement; hard labour; poor living; damp room for half the day. | 12. Confinement; heated close atmosphere; admission of foreign matter into the lungs; constant upright position; wet feet and person, in wet spinning; hurried eatings; sudden transitions of temperature; accidents from machinery; syphilitic taint; want of cleanliness; gas lights in closed rooms in winter; personal labour only source of support; attention obliged to be as unremitting as the motion of a steam-engine." |

Amongst the hand-loom weavers, human exertion appears to be pushed, in every age of life, to the very utmost limits of capability; and yet the greatest amount of labour which an individual can furnish is barely sufficient to procure, in exchange, the coarsest raiment, the meanest dwelling, and but a stinted allowance of the cheapest food. The following example, taken from one of the Glasgow Reports, is painfully illustrative of this fact.

" John Harrup works in a back damp earthen-floored shop, and sleeps in a miserably dirty garret in the same building; no bedstead, scarcely any furniture. Earns on an average 6*s*. per week, out of which he pays all his loom expenses, more than 1*s*. per week. He is twenty-five years of age; his wife twenty-one; one child; likely soon to have another. He is thin, pale, hollow-cheeked, and looks half-starved. He works from five to nine now, and often longer in winter. Solemnly assures me that he never takes thirty minutes to all his meals, during working hours. Would like exceedingly to become a power-loom dresser, but it requires great interest to get such a berth." 42.

Were we to quote all that is interesting in the paper before us, we should leave ourselves no space to examine the Reports of the other three District Medical Commissioners. Compelled, therefore, to pass over all the curious details connected with the cotton factories of Glasgow, New Lanark, Catrine, &c. the domiciliary visits to the families of cotton-spinners, weavers, colliers, dyers—the examinations of factory invalids of all ages (with the lists of their diseases), presented by the trades-unions in all the large towns, &c, we shall confine ourselves, for the present, to the following extracts from Sir David's own conclusions, in which he contemplates all descriptions of factory labour.*

---

* The Editor having consigned the examination of this report to a gentleman of distinguished talents, and who had extensive knowledge of factory labour, both in England and Scotland, did himself peruse both the review and the original documents on which the review is founded. The Editor cannot allow this article to go forth to the public without expressing his opinion, that the labours of Sir David Barry are underrated by the reviewer. They form a monument to

"With regard to one of the important elements of health, viz. income, there is no doubt that the adult cotton-spinner is better circumstanced than any other class of operatives connected with the manufacture of wool, cotton, or flax; and as to the infant operatives, the comforts of the widowed mother, or indigent parents, are in direct proportion to the number of their children for whom they can procure factory employment.

But, although both the young and the adult mill-workers may command more abundant food and better clothing than their unemployed neighbours, there are causes to whose operation they are exposed, which, in a sanitary point of view, counterbalance the advantage alluded to.

1. The first and most influential of all is the indispensable, undeviating necessity of forcing both their mental and bodily exertions to keep exact pace with the motions of machinery propelled by an unceasing, unvarying power.

2. The continuance of an erect posture for periods unnaturally prolonged and too quickly repeated.

3. The privation of sleep." 72

"Females are much less deteriorated in their appearance by mill-work than males. Amongst some thousand young women whom I have now carefully observed both in and out of their factories, and after having examined upon oath those who had known them longest as to the existence of deformities amongst them, I have not met with one distorted or narrow pelvis. If there be any difference between factory and other adult girls relatively to that portion of the female form, I would say, that in the former, in this country, it is more fully developed. Of all the married women who had been mill-girls from their childhood, whom I visited at their own dwellings, and inquired about from their husbands, there are but two unfruitful. The husbands of all were spinners. The children were numerous for the time the couples had been married, and as healthy looking as those of any class of the community. Spinners almost always marry young, and select girls from seventeen to twenty-two, who immediately quit the mill upon being married; sometimes a little before that event."

"Although all the sources of immediate and prospective suffering, to which I have alluded, may be so far remedied or mitigated by the liberal and benevolent management of large establishments, under enlightened men, as to render twelve hours of factory work compatible with average health and longevity—although I have not ascertained any positive disease or deformity to which factory children are peculiarly liable—although I am sure that the adult male factory operatives and mechanics look forward to an increased demand for their time, from the necessity which they think must arise of erecting new machinery, to compensate for the diminished productiveness which a short-time bill would inflict upon the existing machinery—yet I am of opinion that less labour ought to be required from the infant workers, and that more time should be allowed them for sleep, recreation, and the improvement of their minds, than they at present enjoy. For the reasons already stated, however, the minimum of age for admission to factory work ought not to be raised above the end of the tenth or eleventh year, obliging the employer to ascertain that the child can read before it be employed." 73.

### II.—DR. LOUDON'S REPORT

Occupies 25 pages, and consists, 1st, of a letter from Leicester, mentioning an excursion to some collieries in the country, and quoting a rather vague account given by a person in charge of one of them, which, we are told, is applicable

---

the industry, zeal, candour, talent, and tact of Sir David Barry—a gentleman, between whom and the Editor, the readers of this Journal well know there have been some sharp collisions. But the Editor would do violence to his sense of merit and his wish for justice, did he not take this opportunity of expressing his sentiments respecting the Factory Commission. With the talents, integrity, and high professional acquirements of the other Commissioners, the Editor is well acquainted; but he must say that the industry and tact of Sir David Barry, in this investigation, entitles him to the foremost rank in a laborious and difficult inquiry.

Palmam qui meruit ferat.

to the morality, education, and earnings of the operatives employed in that, and six other collieries. There are some general observations on the manufactures of the town, amongst which the following, if it had been supported by circumstantial evidence, would be important :—" On the whole, it does not appear that the misery portrayed in the evidence before the House of Commons exists, to any extent, in Leicester." We regret to say that no personal examinations—no individual cases are recorded.

2. In a letter from Nottingham, where five factory-workers are stated to have been examined—two of them were deformed; one was a boy " with twisted knees, owing to long standing in the mill"—the other was a female, aged 45, who " commenced working in factories when five years old." There is nothing said as to the nature or seat of her deformity. It afterwards appears, however, from Dr. Loudon himself, that the deformity, whatever it was, was not in the pelvis.

There is a remark which we quote, from its agreeing with the interesting observation of another Medical Commissioner, as to the effect of an habitual exposure to a very high temperature on the health of young females :—" Visited the drying-room of Mr. Spencer, but never did I see so many healthy young women together, although the temperature was about 85°."

Here we would be led to suspect that the examination, in this instance, was not made with sufficient care, the degree of heat employed in drying-stoves being, we think, underrated. Sir David Barry, in his report on the stoves, at the great bleaching and dyeing establishment of Mr. Monteith, of Glasgow, states that Fahrenheit's thermometer rose in his own hand to 140°, whilst he stood in the midst of the stove-girls at their work, and that these girls were all in good health, although constantly passing through the open air from one stove to another ; and that, when any of them happened to catch cold, they were very soon cured by going into the stove again.

3. We have next a communication from Leeds, containing notes of the examination of five or six witnesses, who had been previously examined before the Committee of the House of Commons ; and covering detailed formal opinions by ten medical men of Leeds and Bradford. These latter documents occupy about 18 pages, and look very much like the studied productions of their authors. These opinions differ in no small degree amongst each other, as to the effects of factory-labour upon the youthful part of the population of the Leed's district ; some asserting that it produces little or no unfavourable change in the health or form ; others, that twisted bones, broken-down insteps, and cachectic diseases, are its frequent consequences. All, however, or almost all, agree that the hours of infant labour ought to be shortened.

### 4. *Conclusions by Dr. Loudon.*

These, as far as they relate to the effects of factory labour upon the mill-workers, are contained in a very small compass, and are chiefly founded upon such of the written statements already alluded to, as denounce the morbid effects of that labour in the strongest terms. " No cases presented themselves of deformed pelvis, varicose veins, ulcers in young people under twenty," &c. Upon this statement we cannot help remarking, that deformed pelvis must be a species of factory lesion of extreme rarity, because it would " present itself," even to the most superficial spectator, for it cannot be concealed whenever it exists ; but varicose veins, and ulcers in the lower extremities, can only be detected by careful examination, for they do not obtrude themselves on our notice, and are most studiously concealed by the young adult female. Their frequency and dangerous magnitude however in mill-workers, are not the less certain, as may be learnt by referring to the numerous and frightful cases of enlarged veins seen by Sir D. Barry, who states that he found those whose work obliged them to stand constantly, more subject to varicose veins of the lower extremities, and to a larger and more dangerous extent than he had ever witnessed even in foot-soldiers.

There are several administrative and financial suggestions thrown out by Dr. Loudon in his concluding remarks, which would have the effect of creating and placing in the patronage of Government certain appointments to be most advantageously filled by medical men, more especially by such as, from having been educated in manufacturing places, must be intimately conversant with the arrangements and economy of factories.

Knowing the talents and acquirements of Dr. Loudon, we must say that we were somewhat disappointed in the perusal of his report. We expected one of the most philosophical investigations which the subject would possibly admit of; and we looked for a freshness and originality of remark and observation, of which we well knew him capable—but we have been a good deal disappointed! We have been informed that the Doctor's health was not very good at the time, and this may account for the deficiency of personal labour in his report, as compared with that of Sir D. Barry.

### III.—Dr. Hawkins' Report.

This gentleman was the Medical Commissioner for the Lancashire District; by far the most important in the empire, as regards factory-labour. It was but fair to afford the distinguished author of " medical statistics" an opportunity of visiting Manchester in 1832, a place which he had rendered so strikingly remarkable in 1829 for the very low rate of annual mortality assigned by him to its inhabitants, as compared with those of other towns.

After estimating the annual number of deaths in London, at 1 in 42, Dr. Hawkins, at page 19 of his Statistics, goes on to say—" One city alone, in Europe or in England, approaches to London in the value of life, proportionately to its size; it is the second in England in number of inhabitants, the seat of manufactures—*Manchester*. The mortality of Manchester was, about the middle of the last century, 1 in 25; in 1770, 1 in 28. Forty years after, in 1811, the annual deaths are diminished almost beyond belief, to 1 in 74; but the improvement does not stop even there, for in 1821 they became still fewer, although the population has been quadrupled during the 60 years through which the deaths have so diminished." This improvement of health is attributed, in a great measure, to certain police regulations, and to ventilation. At the very time (1811) that this extraordinary salubrity is given to Manchester, the annual deaths in Liverpool were 1 in 30, and in Birmingham 1 in 34. Now, as Dr. Hawkins's Report, (written in London,) on the sanitary state of the factory population of Manchester in 1832, is but three years later in date than the preceding quotation, it would be no more than natural, that he could not pronounce factory-labour to be frightfully destructive of health, without offering some explanation as to the astounding degree of comparative salubrity which he had so lately found to prevail in the very spot where that labour was at the time, and is now pushed to its very highest tension. Yet such appears to be the fact. Dr. H.'s Report makes no allusion to his " Elements of Medical Statistics," and we are left to reconcile the one with the other in the best way we can.* The following are specimens

* The Editor of this Journal, who is not the reviewer on the present occasion, begs to state not merely his belief, but his certainty, that some most unaccountable error, crept into Dr. Hawkins' original statement as to the ratio of mortality in Manchester. He has been assured by one of the most talented and best informed actuaries in this kingdom, that the estimate is totally erroneous, and that the proportion of deaths in Manchester, so far from being only 1 in 74, is little less than 1 in 40."—*J. J.*

of the Dr.'s conclusions, as to the present state of the public health and mortality of Manchester.

" In order to ascertain the state of health of the youthful factory classes, compared with youth in other conditions, I made a careful examination of the Bennet-street Sunday-school at Manchester, in which abundance of all trades exists. I accordingly took an account of 350 of both sexes not engaged in factories, and of 350 of both sexes engaged in factories. Of the former, several remain at home and do nothing; some are in service, some are dress-makers, some engaged in warehouses and in shops. Their age varied from nine years to twenty for the most part.

| Of 350 not in factories, | But of 350 in factories, |
|---|---|
| 21 had bad health, | 73 had bad health, |
| 88 had middling health, | 134 had middling health |
| 241 had good health. | 143 had good health. |

Again, at the St. Augustine's Sunday-school at Manchester, I compared fifty boys engaged in factories, with fifty boys not in factories, some of whom lived at home doing nothing, while others were engaged in shops and in various trades.

| Of the 50 not in factories, | But of the 50 in factories, |
|---|---|
| 1 had bad health, | 13 had bad health, |
| 18 had middling health, | 19 had middling health, |
| 31 had good health. | 18 had good health. |

It will be seen that the advantage of health is at least double, at these institutions, on the side of those young people who are not engaged in factory work." 2.

" On comparing the number of deaths under two years of age in Manchester, Liverpool, and London, they appear to be far more numerous at Manchester than in the two latter places; of 1,000 persons buried in one year in those three places, 424 die at Manchester under two years of age, 362 at Liverpool, and 308 at London." 5.

We are sorry to find compressed into one short sentence, not only all that our author has thought proper to say on the diseases of the Manchester manufacturers, but also a sort of justification of his conduct in having said no more.

" It would be superfluous to discuss, within my present narrow limits, the peculiar diseases which may or which may not attach themselves to this condition of life, and respecting which some conflict of opinion prevails; but on all sides it is admitted that indigestion, hypochondriasis, and languor affect this class of the population very widely." 4.

The exemption of factories from cholera appears to have been very remarkable.

The information furnished by Dr. Hawkins to the Central Board covers nineteen pages, five of which are the Doctor's own, whilst the other fourteen are composed of parole evidence, answers to set queries, and the written opinions of medical men, not however (we are sorry to say) as to health and disease, but as to the abstract question of *short* or *long* hours of work. In this report we have again to lament the total absence of individual sanitary description, either of factory-buildings, workers, or hygienic circumstances of factory-life. In short, we have no portrait of any person or thing to prove to us, that the artist had carefully studied the original. Unfortunately we have nothing from the spot. The number of hours work, in relation to the age of the worker, occupies the Dr.'s mind exclusively. Personal communication with the Central Commissioners after his return to London, may account for this, and a difference of opinion on the points just mentioned, may have given to the Report before us the polemic colouring it displays, more particularly when contrasted with some of the measures recommended by the Central Commissioners.

### No. IV.—Report by Dr. Woolriche.

The results of this gentleman's investigations in the Western District, having been forwarded to the Central Commission early in his progress and jointly with those of his immediate civil colleagues, do not appear in a separate form. His communications are not voluminous, but they are practical, divided, and comprehensive; such indeed as might be expected from an experienced military medical officer of long standing, independent character, and well disciplined mind; a man accustomed to deal with large masses of human beings under various circumstances of privation and suffering; a man taught by a thousand lessons to examine closely, and to trust chiefly, if not solely, to his own observation in all that is connected with the existence of individual disease. His sanitary picture of "the inhabitants of the beautiful vallies of Devon and Somersetshire," is highly flattering to the manufacturing classes.

"In many of the factories the children have an appearance strikingly healthy; some are unusually ruddy, active, and lively; and I state deliberately, that *I saw no case* of disease or distortion which could in *fairness* be solely attributable to the *employment* of the individual. That persons working in factories are not exempt from their full proportion of disease must be admitted; but after the inspection of many thousand operatives, old and young, I feel convinced, that taking into account the advantages and disadvantages of the manufacturing classes, as regards the confinement on the one hand, and their abundant supply of nourishing food on the other, the balance will be found considerably in their favour, as compared with the badly-paid agricultural labourers of the present day." 15.

Having found no disease produced by the manufacturing of "fine woollen cloth," and having found the manufacturers better off than their agricultural neighbours, he wisely, we think, recommends to let well alone, and to abstain altogether from uncalled-for legislation.

A careful perusal of these Reports, as well as those of the Civil Commissioners, has left the conviction strong upon us, that factory-labour, more particularly in cotton and flax mills, tends not a little to deteriorate both the mind and body in young persons so employed; but certainly not to the extent that Mr. Sadler, Mr. Oastler, and their school would have us believe. Much that is unfavourable to health and to high physical development of the human animal is common to all these classes of the community, whose bodily labour is from their infancy the only source of daily subsistence. It is therefore obviously wrong to attribute all the evils found attendant upon any one class to the circumstances in which that class differs from all others. Truth can be found only in the balance of experience. But can we wonder that enthusiastic philanthropists should have overlooked, or selfish agitators availed themselves of this source of error, when we find certain leading medical men in London (appealed to by the council of the nation) asserting that distorted and unproductive pelves in the female, must be the frequent consequence of factory-labour? The laws of physiology too are quoted by these gentlemen, and accepted in proof of this, as it now appears, most unfounded assertion. What will these high medical authorities say to the stubborn fact, that not one distorted pelvis has been found in all the factories of Great Britain—that abortions and difficult labours are rather less frequent amongst the females of those establishments than amongst those of the other working classes—that the reproductive power is but little, if at all deteriorated? What can they say, in candour, but that, at the time they gave these oracular opinions they had never seen the inside of a factory, nor ever examined the forms of those employed there? But let that pass. There is quite enough of evil without it in the present factory discipline of this country—that vile system which, in every case, converts the chubby playful boy, and the tender interesting girl into so many little locomotive steam engines, with scarcely respite enough allowed from their labour to take in as much of the coarsest fuel as will keep up their moving power. When we find the twisted femur, the cumbered knee, the stunted, degraded form, frequent and direct consequences of this cruel system; whilst the youthful mind, which ought to be a garden of budding flowers, is not only left uncultivated but suffered to rankle overgrown with foul weeds, surely it is time that reform should come, and we are rejoiced to learn that reform has come. May it prove as effectual as the Reports upon which it is founded are honest and unexaggerated.

